# Pioneering low-cost 3D-printed transtibial prosthetics to serve a rural population in Sierra Leone – an observational cohort study

**DOI:** 10.1016/j.eclinm.2021.100874

**Published:** 2021-05-08

**Authors:** Merel van der Stelt, Martin. P. Grobusch, Abdul. R. Koroma, Marco Papenburg, Ismaila Kebbie, Masanga 3D prosthesis printing research group, Cornelis. H. Slump, Thomas J.J. Maal, Lars Brouwers

**Affiliations:** a Masanga Medical Research Unit, Masanga Hospital, Masanga, Sierra Leone; b Masanga Hospital, Masanga, Sierra Leone; c Technical Medicine, University of Twente, Enschede, The Netherlands; d Radboudumc 3D lab, Radboud University Medical Center, Nijmegen, The Netherlands; e Center of Tropical Medicine and Travel Medicine, Amsterdam University Medical Centers, location AMC, Amsterdam Infection & Immunity, Amsterdam Public Health, University of Amsterdam, Amsterdam, The Netherlands; f Papenburg Orthopedics B.V. Ravenstein, The Netherlands; g National Rehabilitation Programme/Centre, Ministry of Health and Sanitation, Freetown, Sierra Leone; h Department of Surgery, Radboud University Medical Center, Nijmegen, The Netherlands; i Department of Surgery, Elisabeth Tweesteden Hospital, Tilburg, The Netherlands

**Keywords:** Sierra Leone, 3D-printing, Low-cost, Transtibial prosthetic socket, Fused deposition modelling, Follow-up

## Abstract

**Background:**

There is a huge unmet global need for affordable prostheses. Amputations often happen in Sierra Leone due to serious infections, complex wounds, traffic accidents and delayed patient presentation to the hospital. However, purchasing a prosthesis is still beyond reach for most Sierra Leonean amputees.

**Method:**

We applied computer-aided design (CAD) and computer-aided manufacturing (CAM) to produce low-cost transtibial prosthetic sockets. In February and March 2020, eight participants received a 3D printed transtibial prosthesis in the village of Masanga in Tonkolili district, Sierra Leone. Research was performed using questionnaires to investigate the use, participants’ satisfaction, and possible complications related to the prostheses. Questionnaires were conducted prior to production of the prosthesis and five to six weeks after fitting the prosthesis. A personal short-term goal was set by the participants.

**Findings:**

Competitively priced and fully functional prostheses were produced locally. After six weeks, all participants were still wearing the prosthesis and six of the eight participants reached their personal rehabilitation goals. Using their prostheses, all participants were no longer in need of their crutches.

**Interpretation:**

We have come a step closer to the production of low-cost prostheses for low-and middle-income countries (LMICs). The goal of our project is to perform long-term follow-up and to refine our concept of 3D printed prostheses for LMICs to provide practical solutions for a global health need unmet to date.

**Funding:**

€ 15,000 was collected during a crowdfunding campaign in collaboration with the Dutch Albert Schweitzer Fund. Internship allowance for MvdS was obtained from the University of Twente. 3D-scanner, 3D-printer, and printing material were donated by Ultimaker BV and Shining 3D.

## 1 Introduction

The International Society for Prosthetics and Orthotics and the World Health Organization have estimated that 0.5% of the population in low-and middle-income countries (LMICs) need prostheses or orthotics [1]. In general, facilities to produce prostheses are not within reach for participants and the production of a prosthesis is too expensive in lower income strata [2].

Amputees have a lower quality of life compared to the general population [2]. When people are discouraged from accessing services, they are often excluded from participating in society [2], [3], [4]. In addition, without access to proper protheses, people develop secondary conditions, which will increase the impact of their disability [5]. It is known that prostheses in general positively affect the quality of life [3,6,7].

Sierra Leone ranks 184 of 187 on the UN development index [8]. Amputations often happen in Sierra Leone due to serious infections, complex wounds and traffic accidents [9]. In addition, a common problem is the delayed patient presentation to the hospital, and an initially small medical problem often leads to irreversible damage in the long term [10]. As in many other countries, reliable information about the number of people in need of a prosthesis is not available, and information about the number of amputees in Sierra Leone is available only to a limited extent. Most recent data available from the Population and Housing Census (PHC) study indicated that Sierra Leone counted 8,305 amputees out of a population of 7 million [11]. However, in 2018, 65 amputations were already performed in the Masanga Hospital alone, of which 44 were lower limb amputations. Also recent data on the number of amputations performed in 2018 in Western Area of Sierra Leone is available. In this area 40 lower limb amputations were performed in public health facilities [12]. Therefore, the possibility exists that the number of people who have been amputated and need a prosthesis may be much higher than is indicated by the PHC.

Directly after the civil war (1991–2002), prosthetic devices were provided free of charge to patients at the rehabilitation centres in the cities of Freetown, Makeni, Bo, and Kono. According to the National Physiotherapy and Rehabilitation Programme, this was done with the help of Handicap International (in Freetown, Bo and Kono from 1996 to 2012) and Prosthetic Outreach Foundation (in Makeni from 2005 to 2010). When these agencies handed over the centres to the ministry of health, people had to pay for the prostheses themselves due to lack of consumables and materials at the centres. Many patients could not afford the costs, which created a barrier to the accessibility of prosthetic devices. A leg prosthesis in Sierra Leone costs around 100–200 USD. Considering the average income of 490 USD per capita per year, purchasing such a prosthesis is still beyond reach for most Sierra Leonean amputees [13].

The Prosthetist/Orthotist scholarship program of the Society for Prosthetics and Orthotics and the United States Agency for International Development (ISPO-USAID) successfully produced 204 graduates from 44 developing countries graduating between 2006 and 2015. Of this number, 81 were graduated in Africa, of which only 1 in Sierra leone [14]. To the best knowledge of the authors, Sierra Lone has currently no cooperation with this organisation.

According to the National Physiotherapy and Rehabilitation Programme in Sierra Leone, four prosthetic and orthotic departments are located in Sierra Leone; in government hospitals in Freetown, Makeni, Bo, and Kono. Seventeen staff members are employed at these departments: seven employees were trained by the Tanzania Training Centre for Orthopaedic Technologists (TATCOT), of which one with a BSc degree, three with a diploma and another three with a certificate in prosthetics. Four employees have a certificate in prosthetics and were trained by Handicap International in Sierra Leone and six people are trained by local staff in Sierra Leone. There is currently no ongoing training of prosthetic staff. Apart from these registered initiatives, there are some private workshops in Sierra Leone; however, detailed information is not available.

Currently, the measurement of the stump and the production of the socket of such prostheses are carried out manually using plaster molds. The socket shape is highly dependent on the experience and skills of the prosthetist; this implies difficulty in quality assurance [15], [16], [17], [18]. There is an increasing interest in exploiting state-of-the-art 3D printing technology for clinical problem-solving; and an alternative way to design and produce prosthetic sockets is using computer-aided design (CAD) and computer-aided manufacturing (CAM) systems [19]. Using CAD-CAM techniques, the production process is consistent and faster. Furthermore, the entire process can be automated; whereby the socket fitting would become less dependent on the individual prosthetist's skills and experience. In that way, the rate of successful prosthetic fitting could increase, especially in LMICs [20].

In collaboration with the 3D laboratory at the Radboud University Medical Centre, a 3D lab was set up in the Masanga Hospital in Sierra Leone [21,22]. In 2018 and 2019, a feasibility study was performed to investigate the use of a 3D printer in a resource-limited healthcare setting [23]. With this relatively simple technology, low-cost 3D-printed arm prostheses and other medical aids were produced within a short time. The study showed that the design and aesthetics were of added value regarding both functionality and restoring confidence.

However, production of leg prostheses was not yet possible. Fused Deposition Modelling (FDM) is a relatively low-cost printing method that was used in our previous study to produce arm prostheses. The main concern with FDM printed sockets was the problem of delamination. As in any layer manufacturing process, the strength of the FDM socket depends on how well one layer of material is bonded to another. FDM printed prosthetic leg sockets are rarely made, and limited research has been performed regarding the strength, comfort and long-term tear, and wear of these devices [24], [25], [26]. In an earlier study, the strength of these low-cost 3D-printed transtibial prosthetic sockets was investigated and evaluated [27]. Tough polylactic acid (tough PLA) was the most suitable print material, due to the high layer-on-layer binding. Using this material, strong transtibial prostheses were created, that almost comply with International Standard for Structural Testing of Lower Limb Prostheses (ISO 10328). The socket successfully completed 2.,27 million steps during the endurance test with a maximum compressive force of 1200N [28]. A maximum load of 6700N was endured by the prosthesis without fracture [28]. To pass the test, three million steps are required.

We hypothesized that the 3D printed prosthetic sockets were sufficiently durable to test in practical life. In this study, further clinical research is conducted to test the low-cost 3D-printed transtibial prosthetic sockets in a rural area of Sierra Leone.

## 2 Methods

During a period of two months, from February 2020 until March 2020, participants received a 3D printed transtibial prosthesis in the Masanga Hospital. All participants included in the study lived in the village where the hospital is located.

The manuscript adheres strictly to the STROBE guidelines for reporting observational studies.

### 2.1 Ethical considerations

The protocol was reviewed and approved by the Scientific Research Committee of the Masanga Medical Research Unit (MMRU). Ethical approval for this study was obtained from the Sierra Leone Ethics and Scientific Review committee, obtained on 6 January 2019. Written informed consent was obtained from all participants for participation in the study and also for publication of photos, videos, and data in which they wear medical aids.

### 2.2 Inclusion criteria

Participants were eligible for transtibial prostheses if they were not in possession of a prosthesis, or when an old prosthesis needed to be replaced. Participants were 18 years of age or older. Participants were not eligible when any wounds were present on the stump, or when the stump was painful. Finally, the participant had to be able to walk independently with crutches. In case of fluid retention within the stump, the stump needed to be bandaged for a week before measuring the prosthesis.

### 2.3 Questionnaires

To test the use, participant satisfaction with the prosthesis, and possible complications due to the prosthesis, research was carried out doing interviews using purpose-developed questionnaires (see Supplementary Online Material 1a-c for the questionnaires applied). The interviews were conducted before obtaining the prostheses, and after a follow-up period of six weeks. Further longer-term follow-ups are planned at six months and beyond. Questionnaires consisted of different categories: current level of mobility, overall impression of the prosthesis, usage characteristics of the prosthesis, and possible complications. In addition, a personal goal was set by the participants. They were asked what they wanted to achieve with the new 3D printed prosthesis.

### 2.4 Prosthetic socket production

**Fig. 1:**
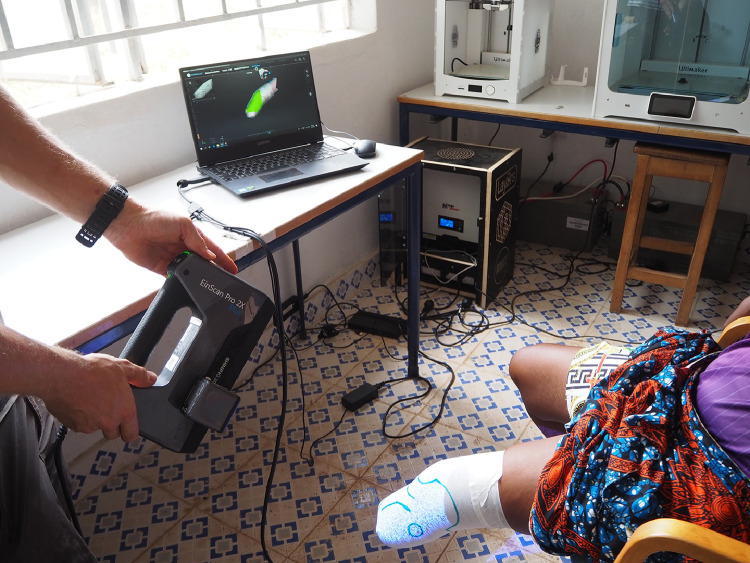
Scanning of the stump with the Einscanner Pro Plus.

An elastic stocking and tight plastic foil were wrapped around the stump to apply extra pressure to the stump and to bind all soft tissue together. The anatomy with the pressure-tolerant and -non-tolerant areas were drawn on this stocking. Later in the design process, these drawings were used to compensate for pressure spots. Dimensions of the stump were scanned with a texture handheld 3D scanner (Einscanner Pro Plus®, Shining 3D Technology, Hangzhou, China) (Fig. 1). A custom-made socket was designed in a free-to-use 3D software tool (Autodesk Meshmixer 3.5, Toronto, Canada) [29], [30], [31].

**Fig. 2:**
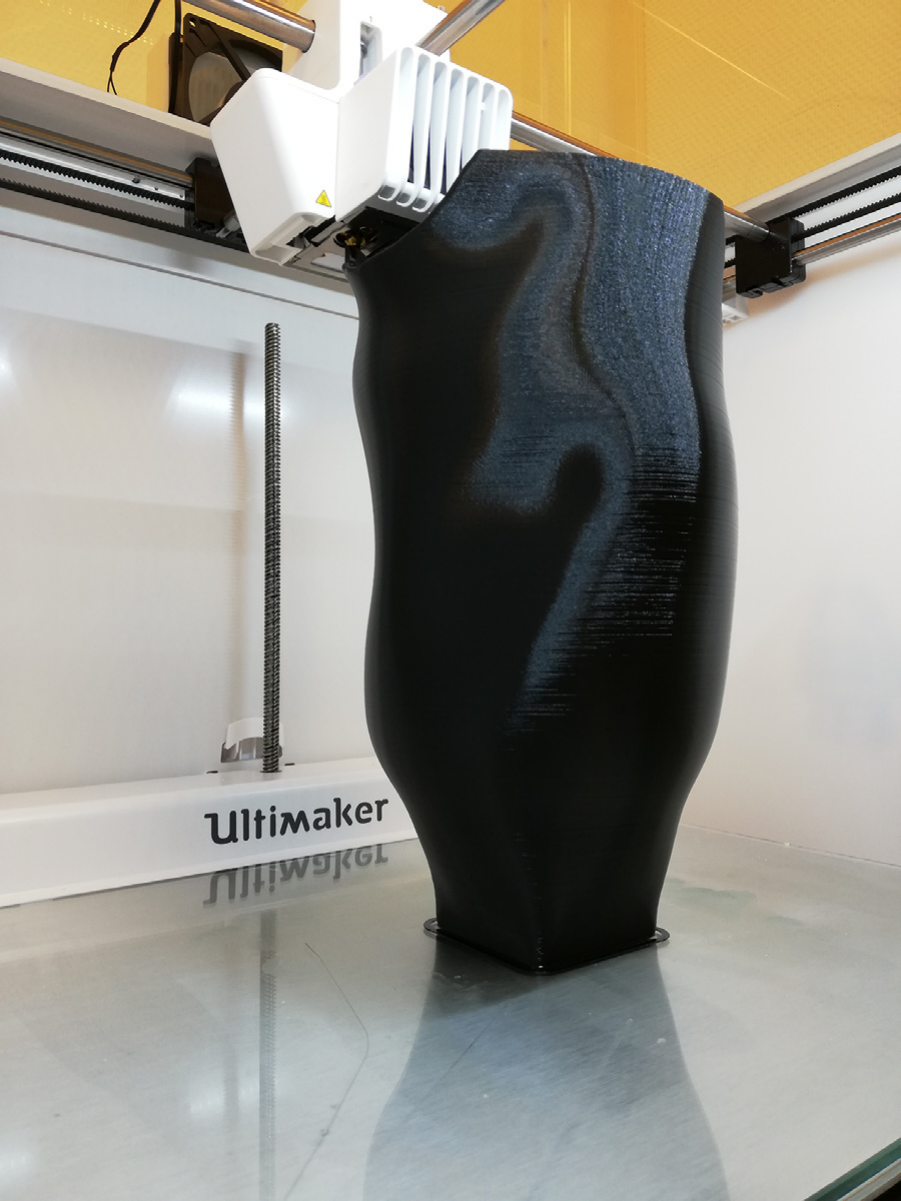
3D printed socket.

Sockets were printed using an Ultimaker S5 (Ultimaker BV, Geldermalsen, the Netherlands), located at Masanga Hospital (Fig. 2) [32]. FDM was used as 3D printing technique. Tough PLA (Ultimaker BV, Geldermalsen, the Netherlands) was used as print material [33]. Before printing, the virtual 3D models were loaded in Cura (Ultimaker BV, Geldermalsen, the Netherlands), used as an open-source 3D printer slicing application, to create a transfer of the 3D model into a printable file (g-code). The socket was printed with a 0.8 mm print core, 100% infill, 0.2 mm layer thickness, and a print speed of 45 mm/sec [34,35].

An off-grid inverter (MPP Solar Inc., Taipei, Taiwan) combined with two 12V/220Ah deep cycle gel batteries (Victon Energy B.V., Almere, The Netherlands) was used to supply voltage to the printer and prevent power changes and outages. The power supply made it possible to print over night when no electricity facilities were available.

### 2.5 Fitting and alignment of the prosthesis

**Fig. 3:**
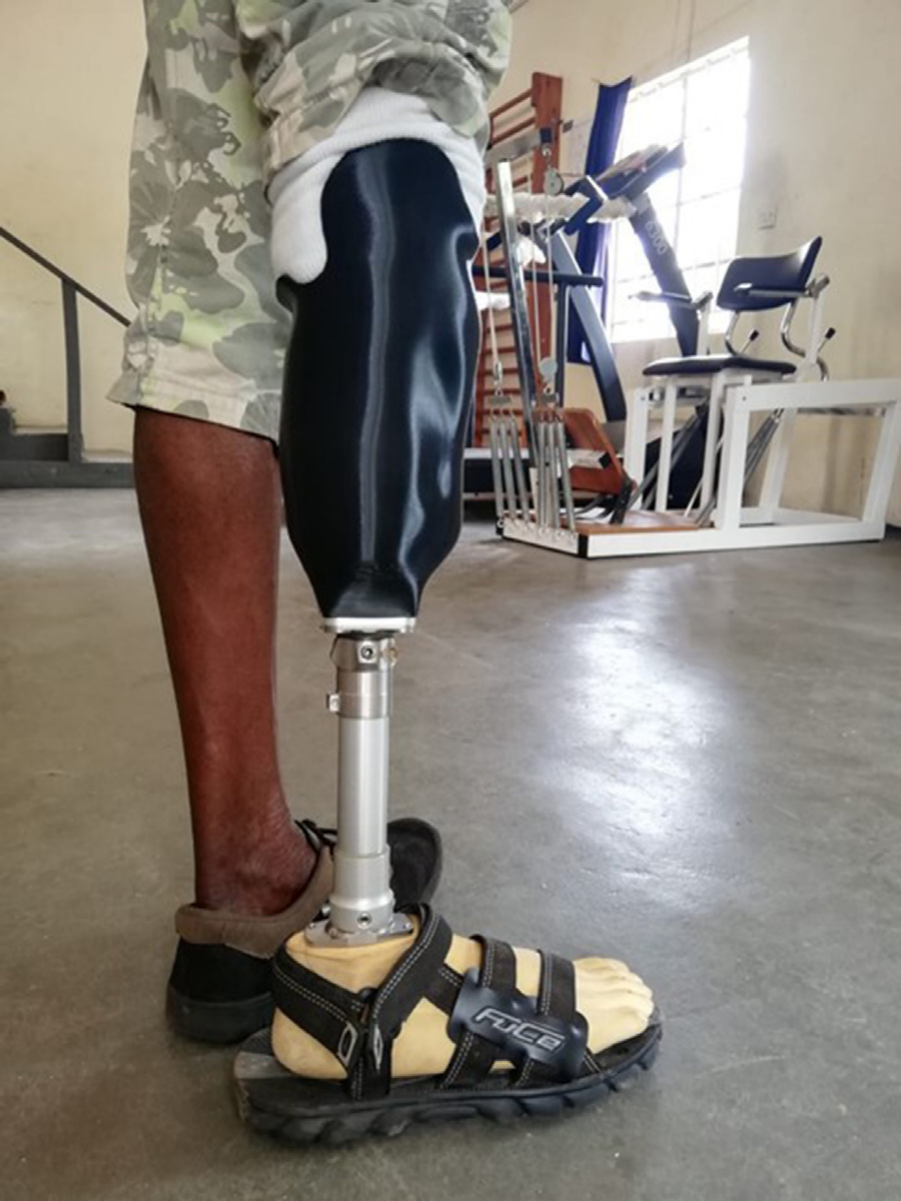
Prosthesis with 3D printed socket.

For the attachment of every 3D printed prosthesis, a patella strap was made from cow leather, to prevent the socket from falling off the stump. The pyramid adapter, tube, tube adapter, and the foot adapter were ordered from Simay Medical (Simay Medical Prosthetics Orthotics Ltd., Izmir, Turkey). The prosthetic foot was carved from local wood, made in the size of the patient's foot. All prosthetic parts were attached to each other according to the Otto Bock guidelines for alignment of transtibial modular lower limb prostheses [36]. Finally, minor adjustments were made to prevent pressure spots by bending the thermoplastic slightly using a 1500W heater. All these steps were performed locally in Sierra Leone. The final prosthesis is shown in Fig. 3.

### 2.6 Physiotherapy

In this study, a similar rehabilitation process was followed as the protocol used at the Radboud Medical Center in Nijmegen, The Netherlands, but adapted to an African setting. The detailed adapted rehabilitation process is shown in Table 1. The participants were asked to participate in physiotherapy for two hours a day for a period of two weeks, depending on the participant's improvement of the rehabilitation.

**Table 1:** Participant rehabilitation programme outline.

Learning to put on the prosthesis Symmetrical posture training Pelvic shift exercise Active pelvic lift exercise First phase of the step with the prosthetic foot Swing phase exercise Walking in the bridge Walking with 2 crutches in 3-point pass Walking with 2 crutches in 2-point gait Walking with 2 poles in 2-point gait Walking with Nordic walking poles Walking without a tool Walking with different walking speeds Walking on uneven terrain Various walking shapes: sideways, backwards, stopping and turning Walking up and down slope

If the prosthesis was considered satisfactory by both the participant and healthcare worker, and the participant was able to walk without a tool or safely with crutches, the participant was allowed to take the prosthesis home. The participant returned to Masanga Hospital twice a week to evaluate the ongoing rehabilitation process.

### 2.7 Aesthetic appearance of the prosthesis

**Fig. 4:**
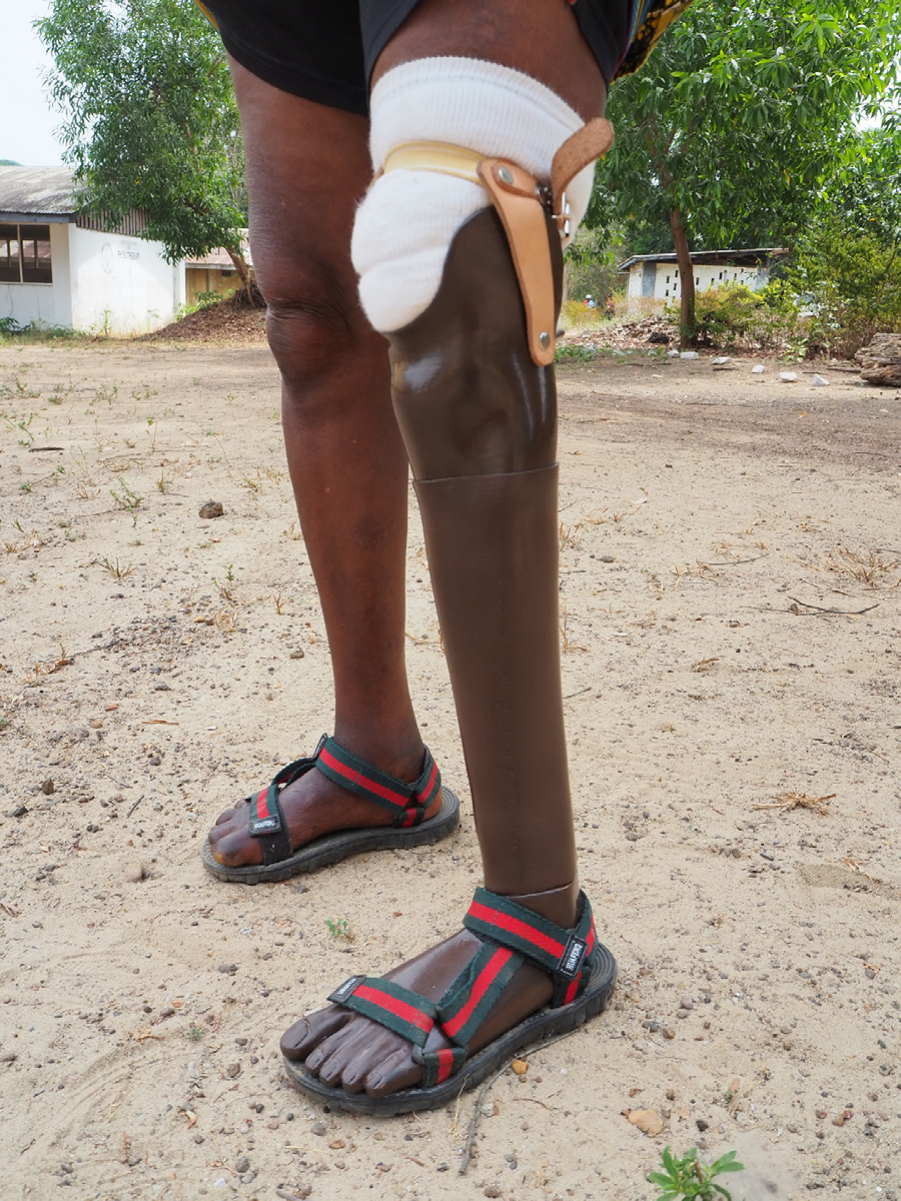
3D-printed prosthesis with aesthetic coverage.

At the final stage of the rehabilitation process, a coverage was created to provide an aesthetical finish around the pyramid adapter, tube, and the foot adapter. To protect the prosthesis from dust, moisture, and ultraviolet light, the prosthesis was coated with Epoxy, (XTC-3D^TM^, Smooth-on, Macungie, Pennsylvania, USA) [37]. To provide the prosthesis with a brown colour adapted to the participant's skin tone, the Epoxy was mixed with UV resistant colour pigment (UVO^TM^, Smooth-on, Macungie, Pennsylvania, USA) [38]. Fig. 4 shows the colored 3D-printed prosthesis, including the aesthetic coverage, as worn.

### 2.8 Funding

In autumn 2019, € 15,000 was collected during a crowdfunding campaign in collaboration with the Dutch Albert Schweitzer Fund (NASF). With this amount, an MPP Solar off-grid inverter combined with two 12V/220Ah Victon Energy gel batteries were financed to prevent power changes and outages of the printer. 3Prosthetic materials were also funded from this grant. We support local employees with a small salary contribution for their indispensable work, and a research 4-wheel drive car was donated to the Masanga Medical Research Unit.

An internship allowance for Merel van der Stelt was obtained from the University of Twente for covering costs of ethical approval, and transport.

The 3D-scanner, 3D-printer, and printing material were donated by Ultimaker BV (Geldermalsen, the Netherlands) and Shining 3D Technology (Hangzhou, China).

The funding bodies had no role in project planning, data collection, analysis and interpretation of data, and the writing process.

## 3 Results

**Fig. 5:**
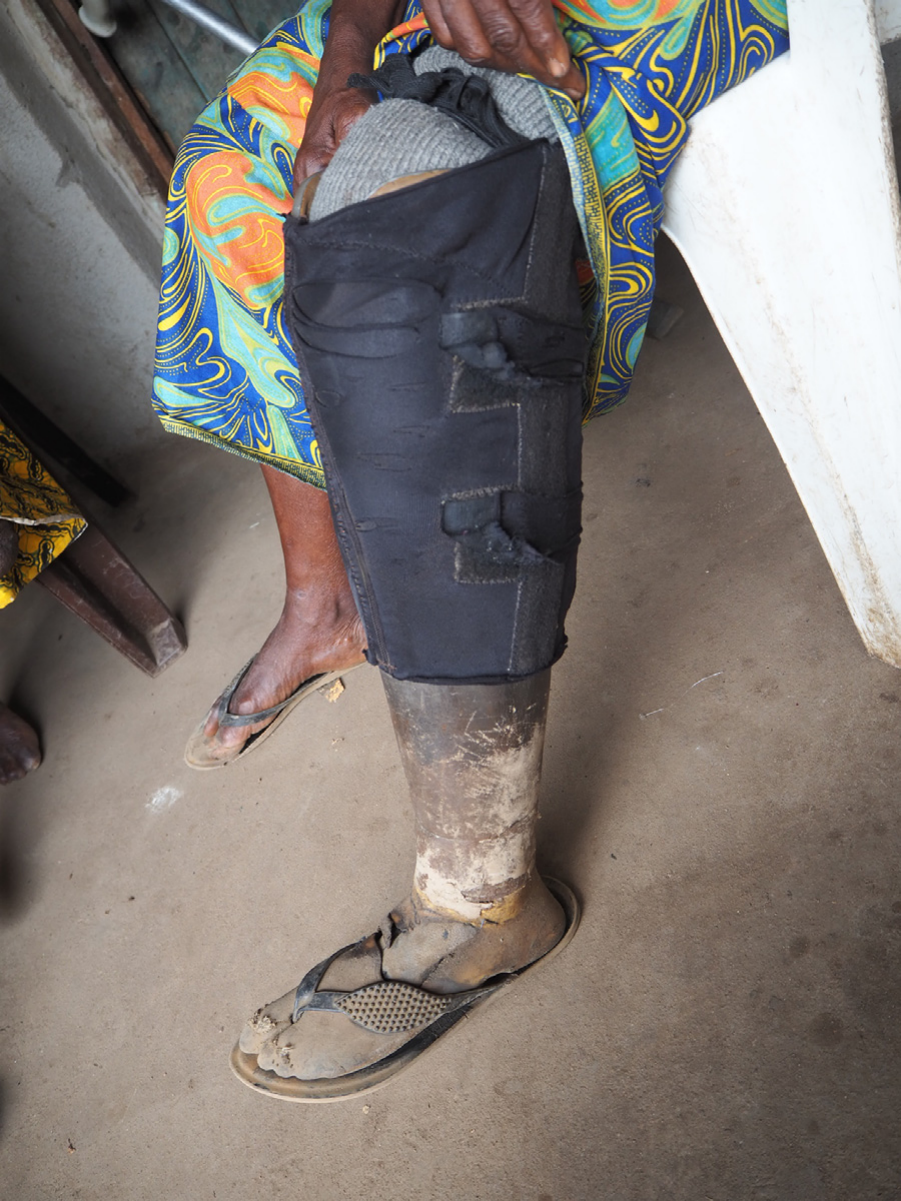
Example of an old prosthesis.

Although a total of twelve participants were plannend to include in this study, eight transtibial prostheses were made before the project had to be temporarily discontinued due to the evolving COVID-19 pandemic. An overview of the characteristics of the participants is presented in Table 2. Three out of eight participants were having a regular prosthesis before receiving the 3D-printed prosthesis, of which two participants were still wearing the old prosthesis at the moment of inclusion. These prostheses were all broken at the foot, and the sockets was too spacious. The stumps were covered with multiple socks, and bandages to fill the socket. An example of an old prosthesis is shown in Fig. 5. For all participants, the main reason for the lack of prosthetic rehabilitation were the high costs.

**Table 2:** General participant characteristics.

Age (years)	43.1 (SD: 15.4)
Gender (male:female)	3:5
Elapsed time after amputation (years)	5.6 (SD: 5.5)
Cause of amputation	3/8 leprosy
	3/8 chronic ulcer
	1/8 tropical ulcer
	1/8 accident
Education	6/8 none
	1/8 elementary school
	1/8 middle School
Daily job/activity	4/8 housewife
	1/8 weaver
	1/8 tailor
	1/8 business
	1/8 none

**Fig. 6a:**
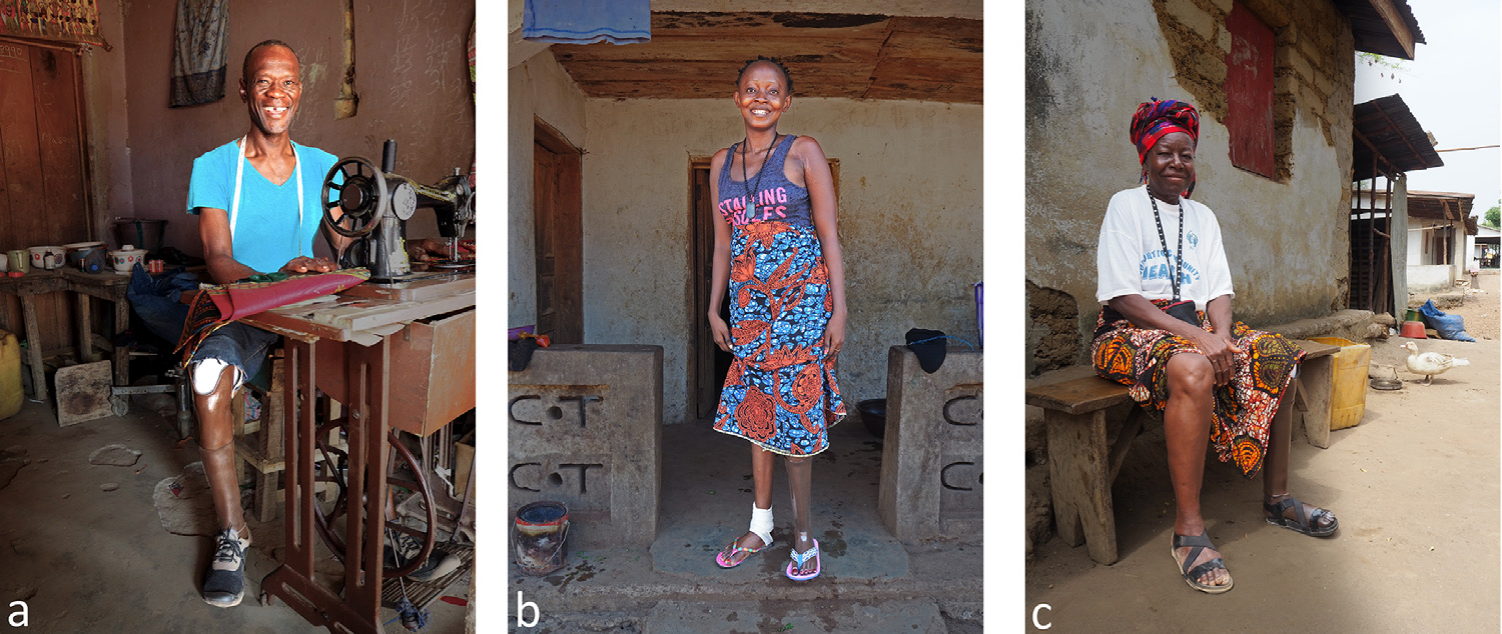
b, c. Result after having received the 3D printed prosthesis.

Table 3a-b provides a summary of the results from the questionnaires (see Supplementary Online Material 2a-c for a more detailed version). One participant deceased during the short-term follow-up period due to a heart attack considered unrelated to the study. During short term follow-up, all participants were still wearing the prosthesis. Six out of seven participants reached their personal goals (Table 3a). One participant indicated slight stump pain, which was related to getting used to the new socket and walking too much. Another participant had a small pressure spot after one week walking with the prosthesis. The pressure spot disappeared after heating the plastic using a heat gun and remodelling the socket. At the end of follow-up, all participants indicated that aesthetics were just as important as functionality. Using their prostheses, all participants were no longer in need of their crutches, an achievement which contributed significantly to the improved well-being of the recipients (Fig. 6a-c).

**Table 3a:** Personal short-term goals set by the participants themselves about what they wanted to achieve in terms of mobility regained with the new 3D printed prosthesis.

Study ID:	Goal to achieve with the new prosthesis:	Achieved: Yes/no

1	Walking without crutches for +/- 400 m. And he wants to be able to operate the weaving machine by foot again.	Yes
2	Walking without crutches for +/- 200 m.	Yes
3	Walking without crutches for +/- 200 m.	Yes
4	Walking without crutches in and around house, walking with crutches outside the house +/- 300 m. And he wants to be able to operate the sewing machine again.	Yes
5	Walking without crutches in the village (+/- 400 m).	Yes
6	Walking without crutches in the village (+/- 400 m).	Yes
7	Walking without crutches in and around the village (+/- 500 m)	No
8⁎⁎	Walking with one crutch around the house (+/- 10 m)	n/a

⁎⁎ Deceased during follow-up period (death not related to intervention).

**Table 3b:** Comparison of the level of mobility, before and after receiving the 3D printed prosthesis. Also use of the 3D-prosthesis in days/week and hours/day is mentioned. Two measurement moments are at the moment of inclusion at the pre-prosthetic phase, and the follow-up after 5-6 weeks when the participant wore the 3D printed prosthesis.

		Study ID:							
		1*	2*	3*	4	5	6	7	8⁎⁎

Which walking support do you usually use when walking?	Inclusion	Two crutches	Two crutches	Two crutches and old prosthesis	Two crutches	Two crutches	Two crutches	Two crutches	Two crutches
	Follow-up	3D prosthesis	3D prosthesis, for long distance guidance of one walking stick	3D prosthesis, for long distance guidance of one walking stick	3D prosthesis and guidance of two crutches	3D prosthesis, for long distance guidance of two crutches	3D prosthesis, for long distance guidance of one crutch	3D prosthesis and guidance of two crutches	n/a
How many meters can you walk without a break?	Inclusion	100–500	50–100	50–100	< 50	100–500	50–100	50–100	< 50
	Follow-up	500–1000	50–100	100–500	< 50	100–500	100–500	< 50	n/a
How many meters do you walk in one day?	Inclusion	50 - 200	50 - 200	50 - 200	< 50	200 - 500	200 - 500	< 50	< 50
	Follow-up	500 - 1000	50 - 200	50 - 200	50 - 200	200 - 500	200 - 500	50 - 200	n/a
How many days a week do you use the prosthesis?	Follow-up	> 5	> 5	> 5	> 5	> 5	> 5	3	n/a
How many hours a day do you use the prosthesis?	Follow-up	> 10	> 10	> 10	0-2	> 10	8 - 10	0 - 2	n/a

⁎ Participants who had a 3D prosthesis before, of which only participant number 2 and 3 was still using the old prosthesis at the moment of inclusion.

⁎⁎ Deceased during follow-up period (death not related to intervention).

### 3.1 Costs and manufacturing time

It took approximately 15 min to scan the participant's stump; including preparation of the scanner and clinical inspection of the stump. The design of the socket was created using Meshmixer within 20 minutes. A transtibial socket with 4 mm wall thickness was fabricated using the Ultimaker S5 in 17.2 hours (SD:1.9). The 3D-printed sockets weighted on average 352 grams (SD 35). This corresponds approximately to 20 USD filament costs (excl. VAT). The imported prosthetic parts ordered at Simay Medical costed 62 USD (excl. VAT). The local manufacturing of the prosthetic foot costed 5 USD. The entire material costs of the prosthesis were 87 USD.

### 3.2 Weight of the prostheses

A 3D printed transtibial prosthesis weighed avarage 1,560 grams (SD: 78). This is less compared to the conventional local prostheses, which weighed avarage 1,945 grams (SD: 148). Participants who previously wore a conventional local prosthesis indicated that the lighter prostheses had a great advantage regarding wearing comfort.

## 4 Discussion

In this study, clinical research was conducted to investigate the short-term follow-up of low-cost 3D-printed transtibial prosthetic sockets in a rural area of Sierra Leone. At 6 weeks follow-up, all participants were still wearing the prosthesis. The majority of the participants reached their personal goals in terms of mobility. Three participants previously wore an old prosthesis. After rehabilitation, all participants were no longer in need of their crutches.

Similar examples of FDM printed prosthetic legs have been set up in recent years [39], [40], [41], [42]. However, due to the limited research and follow-up performed on these projects, it is difficult to assess the quality of the medical aids. This shows that there is a need for a follow-up study to investigate this technology.

### 4.1 Comparison with the standard for LMICs

Conventional patellar-tendon-bearing (PTB) transtibial prosthetic sockets are widely-used in LMICs [43]. Prosthetics produced by the International Committee of the Red Cross (ICRC) use this technique. Their workflow is considered as the standard for LMICs [20,16,17]. The measurement and production of these sockets are carried out manually [20,44,45]. However, previous research showed that the quality of these prosthetic sockets was at the lower end of the acceptance level [[16], [17], [18],^46^]. Because the positive mold is adjusted manually in the process of PTB sockets, the socket shape is highly dependent on the experience and skills of the prosthetist, with the consequence that quality assurance cannot be given [15], [16], [17], [18].

Using CAD-CAM techniques, the production process can be made consistent and faster [19]. In addition, there is a possibility to automate the entire design process of the prosthetic socket. This should make socket fitting less dependent on the skills and experience of the individual prosthetist, which could increase the number of successful prostheses, especially in LIMCs [20]. In addition, we believe that a standardization of the designing process makes it possible to learn more people this technique in a short period of time. The next few years, an automated designing programme will be developed. MoreF research will be conducted to investigate the the socket comfort of a 3D printed prosthetic socket, produced by 3D-scanning and 3D-designed using an automated designing programme. This research will provide information on the effectiveness of digital produced prosthetic sockets compared to the traditional manufactured prosthetic sockets.

### 4.2 Questionnaires

We collected in-depth data from the participants’ perspectives on their experiences as a prosthetic user in a rural setting. Research was done using self-made questionnaires, since no suitable validated questionnaires were found for testing transtibial prostheses in rural areas. In Sierra Leone, 57% of the adults areilliterate and many people have difficulty interpreting (healthcare-related) questions [47]. Questions should be therefore as simple as possible. For example, estimating distances and time is often difficult. In addition, part of our target group did not speak English, which makes it difficult for the Dutch principal investigator to speak directly with some of the participants. Questionnaires were therefore completed together with local physiotherapists, who are part of the research team and speak all local languages. Distances were estimated based on distance estimations. For example, participants were asked to which village they could walk. This wasconverted into a distance which was completed in the questionnaire. For further research we are investigating the possibilities of collecting data by performing physical tests. Language barriers, different culture and illiteracy will no longer become a barrier to collecting reliable information.

### 4.3 Importance of aesthetics in prosthesis production

At the start of the 3D printing Sierra Leone project, we quickly realised that the demands of Sierra Leonean amputees are different from those in Europe. In our earlier work focusing on arm prostheses, it became clear that aesthetics plays an important role [23]. All participants indicated that aesthetics was just as important as functionality. It was therefore expected that participants would wear the prosthetic leg less often if it would not meet their aesthetic needs. The prostheses were equipped with an aesthetic coverage, so that they resembled a ‘real’ leg in the respective natural skin colour of the individual participant. It was expected that without the aesthetic coverage, the functionality of the prosthesis would be more difficult to test, as the prosthetic would be worn less.

### 4.4 Physiotherapy in remote and resource-constrained settings

In many remote and resource-constrained settings, physiotherapy options are limited [48]. However, physiotherapy is a very important and crucial element of the learning process of walking with a prosthesis. In this study, a two-week rehabilitation process was followed. This is novel to participants in Sierra Leone, where physiotherapy is normally not accessible. During training (see Supplementary Online Material 3 – videoclip), participants indicated a better self confidence in walking and fewer backpain problems. It is therefore recommended to add a physiotherapist into the prosthesis workflow. Involving physiotherapists will become easier in the future, as more students are graduating as physiotherapists in Sierra Leone. In collaboration with the Physiotherapy of Masanga Hospital and the National Rehabilitation Programme, a programme was set up in 2017 to teach students to become physiotherapists. Currently, seventeen students are being educated during a four-year bachelors’ course in physiotherapy at Tonkolili District College of Health Sciences and Technology. The students receive all their theoretical lectures in Masanga and have practical postings at Masanga Hospital and different sites throughout the country.

### 4.5 Cost and import of prosthetic materials

In this study participants were asked to contribute 15 USD. BBy letting participants pay for their prosthesis themselves, a start was made in striving for a sustainable workflow. Many people could not pay this amount up front. Therefore, participants often paid over several weeks. Two of the participants were unable to pay the 15 USD at all, and therefore, the contribution was paid by a different agency. This indicates that (high) costs of medical devices are a major problem in Sierra Leone. Many people are even unable to pay the transport costs to visit a prosthetist [49]. Prostheses must therefore be made at the lowest possible cost to render them affordable for the beneficiary.

Further research will be conducted to replace the imported prosthetic parts by local products, in order to make the project less dependent on imports and to lower the costs even more. We are currently investigating the possibilities of reproducing the comparable prosthetic parts produced by Otto Bock. These parts must first comply with the international guidelines before it can be used in clinical practice. This is important to prevent use of low-quality products, which could result in accidents and injuries. However, this local production of prosthetic parts would only solve a part of the material problem in Sierra Leone. Ideally, it would be best if import of materials could become cheaper and better available in all countries such as Sierra Leone [50,51].

**Fig. 7:**
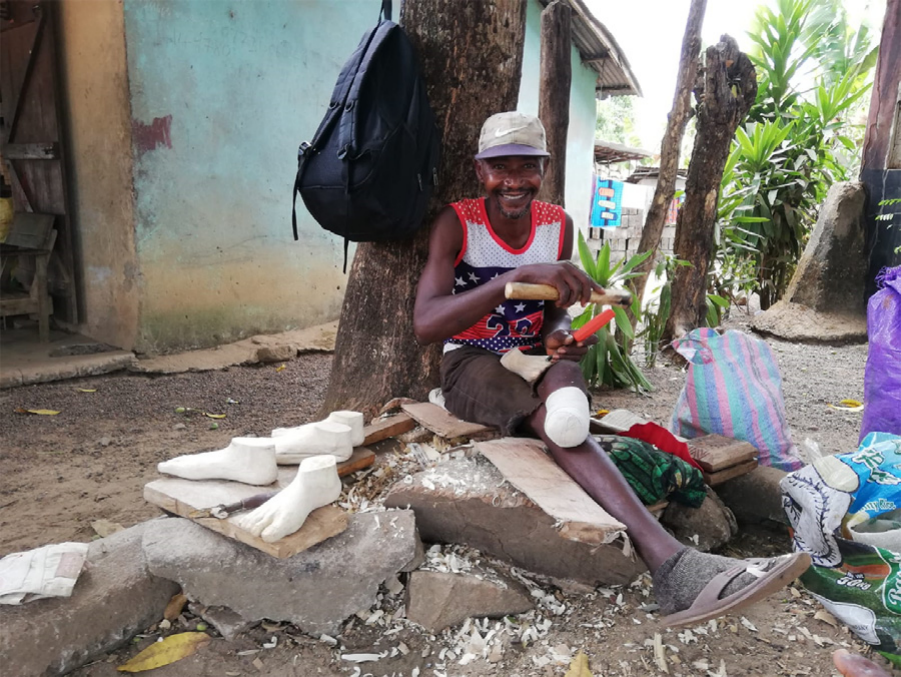
The prosthetic foot is made from wood, in the size of the person's foot, produced by a local hospital patient who is specialized in wood carving.

The conventional prosthetic feet used in Sierra Leone are made of hard material with flexible material at the toeside. After consultation with various patients and local prosthetist, we found out that the foot of the prosthesis often breaks. We have therefore chosen not to use this local foot. The Otto bock basic feet are relatively heavy and have to be imported as well. Therefore, we chose to make the foot ourselves. In the present study, the foot is made of wood by a local woodcarver (see Fig. 7). However, the disadvantage of this foot is that it is still relatively heavy and stiff. The ability to walk on uneven ground and slopes is essential for people in Sierra Leone as very few roads are paved. A less stiff and a light weight foot could help [52]. Further research is currently being done to improve the foot and thereby wearing comfort of a prosthesis.

Based on current estimates, the entire material costs of the 3D printed prosthesis are around 87 USD. These costs are already low compared to the regular prostheses, which costs around 100–200 USD. If the metal prosthetic parts, costing together 62 USD excl. VAT, can be replaced by local products, a further cost reduction is possible. The purchase costs of the electricity supply, computer, 3D scanner and 3D-printer were 11.500 USD excl. VAT. With a depreciation period of five years, the depreciation per prosthetis would be 9.60 USD, if 20 prosthetics are produced per month (11.500 / 5 years / 12 months / 20 prosthetics). A skilled worker costs around 80 USD per month, resulting in 4 USD per prosthesis. Taking this rough calculation into account, assuming that the prosthesis parts and feet can be produced locally for around 20 USD and the printing material costs are 20 USD excl. VAT, a 3D printed prosthesis can be made for around 54 USD. Considering that 3D equipment will become cheaper, these costs may be even lower in the future.

A potential advantage of this technique is that very little tools and materials are needed. A large workshop is therefore not necessary. It would even be possible to create a mobile workshop, so that prosthetic facilities can be available everywhere, even in very remote areas. Technically it would be possible to produce three prostheses within a day, if three 3D printers are available. In this way many people can be helped in a short period of time.

### 4.6 Improved socket strength

Test specifications of all prosthetic components of trans-femoral and trans-tibial prostheses are documented in ISO 10328 [50]. However, there are no standards for testing the prosthetic socket, because this part is custom made. Nevertheless, multiple studies have used these guidelines for testing FDM-printed prosthetic sockets, making this testing method a logical choice to test the prothesis [18,24,53,54]. In recent years, potentially suitable FDM print materials became available with increased layer-on-layer adhesion. This has increased the potential in FDM printed sockets. In earlier study we tested the tensile properties of various potential printing materials suitable for FDM according to ISO 527 (Standard Test Method for Tensile Properties of Plastics). Tough PLA seemed to be the best material, due to the high layer-on-layer binding. During the dynamic test, the prosthetic broke after 2.3 million cycles [27]. To pass the test, three million steps are required. To strengthen the prosthesis, further research is now being performed to improve the socket design and print settings.

### 4.7 Long-term follow-up and proposed further clinical research in Sierra Leone

If the prostheses comply with the International Standard for Structural Testing of Lower Limb Prostheses, if the clinical follow-up in Sierra Leone is successful and if the results of the Dutch study about socket comfort are positive, further research can be done with a larger group of patients in Sierra Leone using the new automated designing programme. The next few years will show whether the benefits of 3D technology will have added value to the production of prosthetics in LMICs.

Competitively priced, fully functional prostheses can be locally produced, and could improve lives of amputees. Furthermore, a locally driven manufacturing programme will create qualified jobs. Long-term follow-up will contribute information about the potential of prostheses for a larger group of people. Designing and printing of the prosthesis is still a process requiring special skills, which are difficult to obtain for people with minor education and no previous ICT knowledge. Automation of the digital designing process can offer a solution for this. In addition, further research will be conducted into replacing the imported prosthesis parts with local products, an improved prosthetic foot and mechanical adjustments to further improve the strengthen the prosthetic socket.

## Author contributions

Conception of the paper (M.v.d.S., L.B., M.P.G.). Field work and data collection at Masanga Hospital (M.v.d.S., A.R.K.). Project oversight and detailed advice regarding the prosthesis manufacturing process (M.P.). Project oversight and detailed advice regarding the national rehabilitation programme (I.K.). Provision of overall technical and medical support (C.H.S.). Provision of technical support in the field of 3D printing in healthcare (T.J.J.M.). Writing of the first draft (M.v.S.). Contribution to the writing, and approval of the final version of the manuscript (M.v.d.S., M.P.G., A.R.K., M.P., I.K., C.H.S., T.J.J.M., L.B.).

Supplementary Material 1a. Questionnaire lower limb prosthesis for participants who have never worn a transtibial prosthesis before.

Supplementary Material 1b. Questionnaire lower limb prosthesis for participants who were already wearing a transtibial prosthesis before receiving the 3D printed transtibial prosthesis.

Supplementary Material 1c. Follow-up questionnaire lower limb prosthesis for participants who received a 3D printed transtibial prosthesis.

Supplementary Material 2a. Overview of general participant characteristics.

Supplementary Material 2b. Data obtained from questionnaire during inclusion.

Supplementary Material 2c. Data obtained from questionnaire during follow-up after 5-6 weeks.

Supplementary Material 3. Physiotherapy session in progress – Videoclip (separate attachment). Physiotherapy is provided by local physiotherapists. In the video you can see one of the participants who have not been able to walk for 20 years. Here he is doing his first steps again with the new prothesis.

## Declaration of Competing Interest

All authors had full access to all the data in the study and accept responsibility to submit for publication. None of the authors has any conflict of interest to declare and all authors have completed the ICMJE form.

Lars Brouwers is Chair of the Foundation 3D printing in developing countries, Merel van der Stelt, Thomas Maal and Martin P. Grobusch are board members of the Foundation. Part of this work constituted a section of Merel van der Stelt's master thesis.

## Appendix Supplementary materials

Image, application 1

Image, video 2
