# Supplementary material for: Pioneering low-cost 3D-printed transtibial prosthetics to serve a rural population in Sierra Leone – an observational cohort study
Source: eClinicalMedicine. 2021 May 8;35:100874. doi: 10.1016/j.eclinm.2021.100874 (PMC8129890; doi:10.1016/j.eclinm.2021.100874)
Supplement: Supplementary file 1 [file mmc1.docx]

## **Supplementary Materials**

# **Pioneering low-cost 3D-printed transtibial prosthetics to serve a rural population in Sierra Leone**

Merel van der Stelt^1,2,3,4*^, Martin P. Grobusch^1,5^, Abdul R. Koroma^1^, Marco Papenburg^6^, Ismaila Kebbie^7^, Cornelis H. Slump^3^, Thomas J.J. Maal^4^, Lars Brouwers^2,8,9^, for the Masanga 3D prosthesis printing research group^‡^

^1^ Masanga Medical Research Unit, Masanga Hospital, Masanga, Sierra Leone

^2^ Masanga Hospital, Masanga, Sierra Leone

^3^ Technical Medicine, University of Twente, Enschede, The Netherlands

^4^ Radboudumc 3D lab, Radboud University Medical Center, Nijmegen, The Netherlands

^5^ Center of Tropical Medicine and Travel Medicine, Amsterdam University Medical Centers, location AMC, Amsterdam Infection & Immunity, Amsterdam Public Health, University of Amsterdam, Amsterdam, The Netherlands

^6^ Papenburg Orthopedics B.V. Ravenstein, The Netherlands

^7^ National Rehabilitation Programme/Centre, Ministry of Health and Sanitation, Freetown, Sierra Leone

^8^ Department of Surgery, Radboud University Medical Center, Nijmegen, The Netherlands

^9^ Department of Surgery, Elisabeth Tweesteden Hospital, Tilburg, The Netherlands

^‡^List of all collaborators at end of paper

* Correspondence: Merel van der Stelt, email: [merel.vandersterstelt@radboudumc](mailto:merel.vandersterstelt@radboudumc).nl

## **Supplementary Materials Legend**

## **Supplementary Material 1a.** Questionnaire lower limb prosthesis for participants who have never worn a transtibial prosthesis before.

**Supplementary Material 1b.** Questionnaire lower limb prosthesis for participants who were already wearing a transtibial prosthesis before receiving the 3D printed transtibial prosthesis.

## **Supplementary Material 1c.** Questionnaire lower limb prosthesis for participants who received a 3D printed transtibial prosthesis.

## **Supplementary Material 2a.** Overview of general participant characteristics.

## **Supplementary Material 2b.** Data obtained from questionnaire during inclusion.

## **Supplementary Material 2c.** Data obtained from questionnaire during follow-up after 5-6 weeks.

**Supplementary Material 3.** Physiotherapy session in progress – Videoclip (separate attachment)

## **Supplementary Material 1a.** **Questionnaire lower limb prosthesis for participants who have never worn a transtibial prosthesis before.**

#### General Characteristics

Name of investigator:

Date:

Study ID:

Age:

Sex:

- Male
- Female

Years/months after amputation:

Level of amputation (TFA, knee-ex, TTA):

Stump length (cm):

Cause of amputation:

Quality of the stump:

Reason not having a prosthesis:

Educational background:

- No school
- Elementary school
- Middle school
- High school
- University & graduate school

Daily activity/job:

#### General questions

1. What is your goal that you want to achieve with the new prosthesis?

- Functional purpose
- Aesthetic purpose
- Functional and aesthetic purpose
- Other:__________________________________________________________

Personal goal:________________________________________________________________

#### Current level of mobility

1. Which walking support do you usually use when walking?

- Walking frame/rollator
- Two crutches or two walking sticks
- One crutch or one walking stick
- Guidance of a person
- None

1. How many meters can you walk without a break?

- Less than 50 meters
- Between 50 and 100 meters
- Between 100 and 500 meters
- Between 500 and 1000 meters
- More than 1000 meters

1. How many meters do you walk in one day?

- Less than 50 meters
- Between 50 and 200 meters
- Between 200 and 500 meters
- Between 500 and 1000 meters
- More than 1000 meters

Additional explanations:_______________________________________________________

**Supplementary Material 1b.** **Questionnaire lower limb prosthesis for participants who were already wearing a transtibial prosthesis before receiving the 3D printed transtibial prosthesis.**

#### General Characteristics

Name of investigator:

Date:

Study ID:

Age:

Sex:

- Male
- Female

Years/months after amputation:

Level of amputation (TFA, knee-ex, TTA):

Stump length (cm):

Cause of amputation:

Quality of the stump:

Reason not having a prosthesis:

Educational background:

- No school
- Elementary school
- Middle school
- High school
- University & graduate school

Daily activity/job:

#### General questions

1. Do you still use the prosthesis?

- No
- Yes

1. What is the reason why you want a new prosthesis?

- The old prosthesis does not fit
- The old prosthesis is broken
- The old prosthesis does not comply with aaesthetic expectations
- Other:__________________________________________________________

1. What is your goal that you want to achieve with the new prosthesis?

- Functional purpose
- Aesthetic purpose
- Functional and aesthetic purpose
- Other:__________________________________________________________

Personal goal:________________________________________________________________

#### Overall impression of the prosthesis

1. How satisfied are/were you with the prosthesis in general?

- Very dissatisfied
- Somewhat dissatisfied
- Somewhat satisfied
- Mostly satisfied
- Very much satisfied

1. How satisfied are/were you with the fit of the prosthesis?

- Very dissatisfied
- Somewhat dissatisfied
- Somewhat satisfied
- Mostly satisfied
- Very much satisfied

1. How satisfied are/were you with the appearance of the prosthesis?

- Very dissatisfied
- Somewhat dissatisfied
- Somewhat satisfied
- Mostly satisfied
- Very much satisfied

1. How satisfied are/were you with the suspension of the prosthesis?

- Very dissatisfied
- Somewhat dissatisfied
- Somewhat satisfied
- Mostly satisfied
- Very much satisfied

1. Does/did the prosthesis give you more confidence?

- The prosthesis gives less self-confidence
- The prosthesis gives little less self-confidence
- The prosthesis does not cause a difference in self-confidence
- The prosthesis gives little more self-confidence
- The prosthesis gives more self-confidence

Additional explanations:_______________________________________________________

#### Current level of mobility in time of wearing the old prosthesis

1. Which walking support do you usually use when walking?

- Walking frame/rollator
- Two crutches or two walking sticks
- One crutch or one walking stick
- Guidance of a person
- None

1. How many meters can you walk without a break?

- Less than 50 meters
- Between 50 and 100 meters
- Between 100 and 500 meters
- Between 500 and 1000 meters
- More than 1000 meters

1. How many meters do you walk in one day?

- Less than 50 meters
- Between 50 and 200 meters
- Between 200 and 500 meters
- Between 500 and 1000 meters
- More than 1000 meters

Additional explanations:_______________________________________________________

#### Use of the prosthesis

1. How many days a week do you use the prosthesis?

- 0
- 1
- 2
- 3
- 5 or more

1. How many hours a day do you use the prosthesis?
   - 0 – 2 hours
   - 3 – 4 hours
   - 5 – 6 hours
   - 8 – 10 hours
   - More than 10 hours

Additional explanations:_______________________________________________________

#### Possible complications relating to the old prosthesis:

1. Do/did you have any skin problems on your stump related to the prosthesis?

- Extremely
- Very
- Moderate
- Slightly
- Not at all

1. Do/did you have stump pain?

- Extremely
- Very
- Moderate
- Slightly
- Not at all

1. Do/did you have phantom pain?

- Extremely
- Very
- Moderate
- Slightly
- Not at all

Additional explanations:_______________________________________________________

## **Supplementary Material 1c. Follow-up questionnaire lower limb prosthesis for participants who received a 3D printed transtibial prosthesis.**

#### General Characteristics

Name of investigator:

Date:

Study ID:

Age:

Sex:

- Male
- Female

#### General questions

1. Do you still use the prosthesis?

- No
- Yes

1. To what extent is the goal that you had described achieved due to wearing the 3D printed prosthesis?

- The goal is achieved
- The goal is somewhat achieved
- The goal is not achieved

1. For what reason do you wear the prosthesis?

- Functional purpose
- Aesthetic purpose
- Both functional and aesthetic purpose
- Other:__________________________________________________________

####

Additional explanations:_______________________________________________________

#### Overall impression of the prosthesis

1. How satisfied are you with the prosthesis in general?

- Very dissatisfied
- Somewhat dissatisfied
- Somewhat satisfied
- Mostly satisfied
- Very much satisfied

1. How satisfied are you with the fit of the prosthesis?

- Very dissatisfied
- Somewhat dissatisfied
- Somewhat satisfied
- Mostly satisfied
- Very much satisfied

1. How satisfied are you with the appearance of the prosthesis.?

- Very dissatisfied
- Somewhat dissatisfied
- Somewhat satisfied
- Mostly satisfied
- Very much satisfied

1. How satisfied are you with the suspension of the prosthesis?

- Very dissatisfied
- Somewhat dissatisfied
- Somewhat satisfied
- Mostly satisfied
- Very much satisfied

1. Does the prosthesis give you more confidence?

- The prosthesis gives less self-confidence
- The prosthesis gives little less self-confidence
- The prosthesis does not cause a difference in self-confidence
- The prosthesis gives little more self-confidence
- The prosthesis gives more self-confidence

Additional explanations:_______________________________________________________

#### Current level of mobility in time of wearing the 3D printed prosthesis

1. Which walking support do you usually use when walking?

- Walking frame/rollator
- Two crutches or two walking sticks
- One crutch or one walking stick
- Guidance of a person
- None

1. How many meters can you walk without a break?

- Less than 50 meters
- Between 50 and 100 meters
- Between 100 and 500 meters
- Between 500 and 1000 meters
- More than 1000 meters

1. How many meters do you walk in one day?

- Less than 50 meters
- Between 50 and 200 meters
- Between 200 and 500 meters
- Between 500 and 1000 meters
- More than 1000 meters

Additional explanations:_______________________________________________________

#### Use of the prosthesis

1. How many days a week do you use the prosthesis?

- 1
- 2
- 3
- 4
- 5 or more

1. How many hours a day do you use the prosthesis?
   - 0 – 2 hours
   - 3 – 4 hours
   - 5 – 6 hours
   - 8 – 10 hours
   - More than 10 hours

Additional explanations:_______________________________________________________

*Possible complications:*

1. Do you have any skin problems on your stump related to the prosthesis?

- Extremely
- Very
- Moderate
- Slightly
- Not at all

1. Do you have stump pain?

- Extremely
- Very
- Moderate
- Slightly
- Not at all

1. Do you have phantom pain?

- Extremely
- Very
- Moderate
- Slightly
- Not at all

Additional explanations:_______________________________________________________

#### Sustainabillity of the prosthesis

1. Did the prosthesis break over time?
   - No
   - Yes
2. How much money is this prosthesis worth to you?

_____________________________________________________________________

1. How much money could you pay for a prosthetic like this?

_____________________________________________________________________

Additional explanations:_______________________________________________________

## **Supplementary Material 2a. Overview of general participant characteristics.**

##

## **Supplementary Material 2b.**


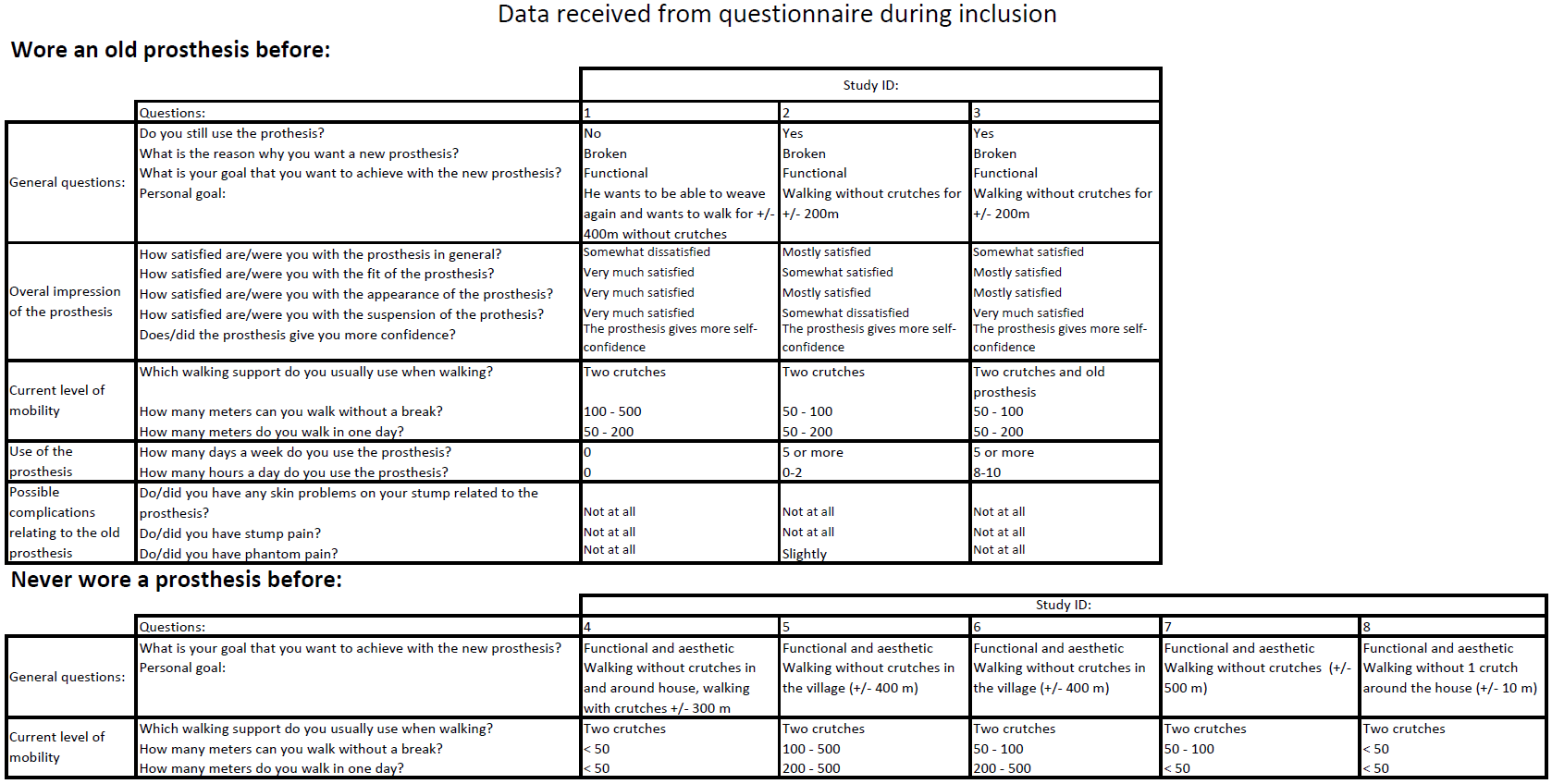


## **Supplementary Material 2c.**


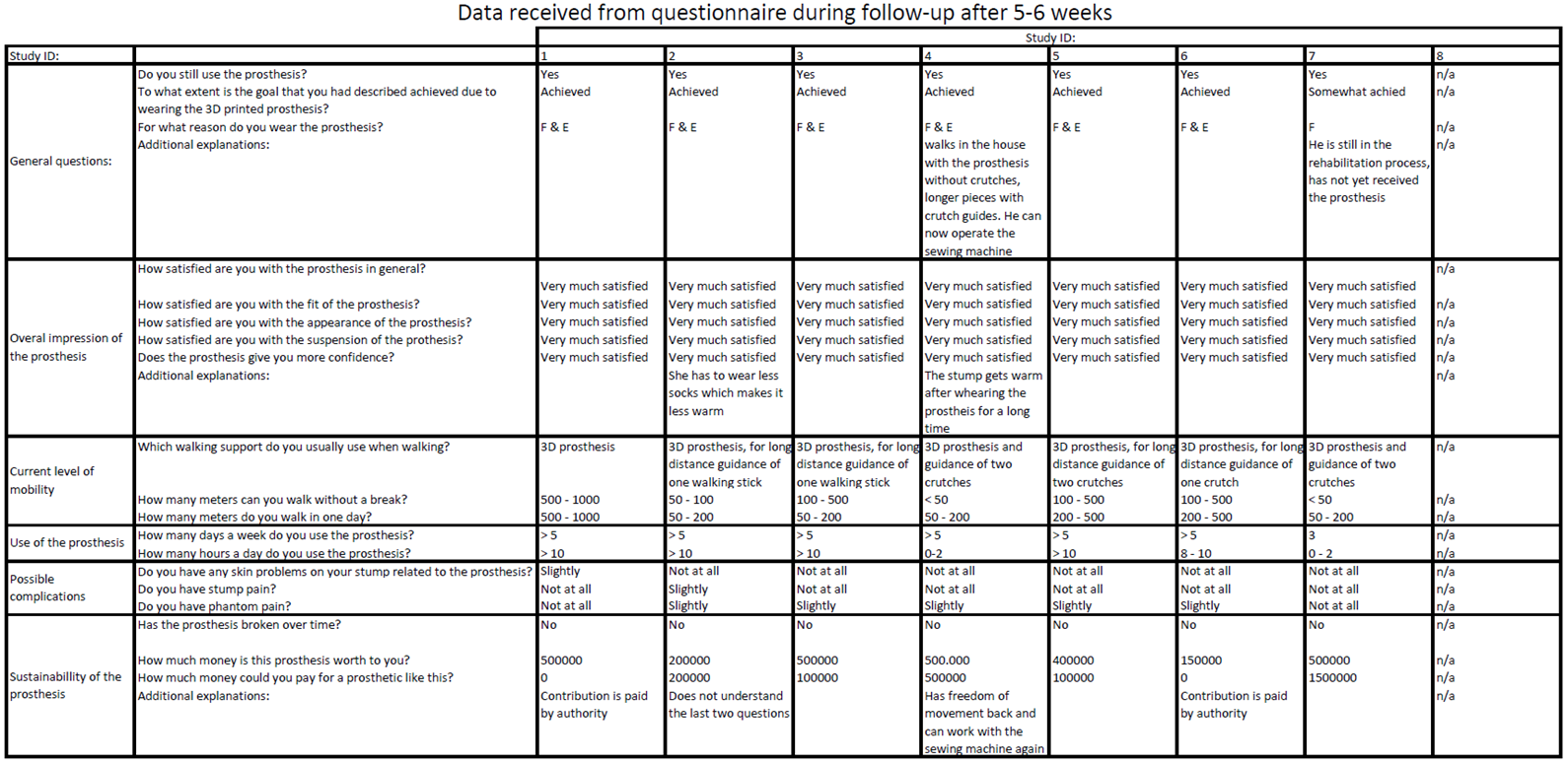


**Supplementary Material 3 – Video Clip**

Physiotherapy session in progress – Videoclip (separate attachment). Physiotherapy is provided by local physiotherapists. In the video you can see one of the patients who have not been able to walk for 20 years. Here he is doing his first steps again with the new prothesis.
